# Comparative Mitogenomic Analyses of Hydropsychidae Revealing the Novel Rearrangement of Protein-Coding Gene and tRNA (Trichoptera: Annulipalpia)

**DOI:** 10.3390/insects13090759

**Published:** 2022-08-23

**Authors:** Xinyu Ge, Haoming Zang, Xiaoyun Ye, Lang Peng, Beixin Wang, Gang Lian, Changhai Sun

**Affiliations:** 1 Lab of Taxonomy & Aquatic Insects, Department of Entomology, College of Plant Protection, Nanjing Agricultural University, Nanjing 210095, China; 2 Environmental Monitoring Station of Qingtian County, Lishui 323999, China; 3 Zhejiang Key Laboratory of Ecological and Environmental Monitoring, Forewarning and Quality Control, Zhejiang Province Ecological Environment Monitoring Centre, Hangzhou 310012, China

**Keywords:** mitochondrial genome, gene rearrangement, *Potamyia*, phylogeny

## Abstract

**Simple Summary:**

The evolution of insect mitochondrial gene rearrangement is a hot topic, and such rearrangements are common in certain insect orders. Gene rearrangement characteristics can also provide effective information for phylogenetic reconstruction. As one of the most diverse families within Annulipalpia, Hydropsychidae Curtis, 1835 is distributed on all continents except Antarctica. Here, we generated 19 novel mitogenomes of hydropsychid species, and found two new mitochondrial gene rearrangements. Coupled with published mitogenomes of Hydropsychidae, we analyzed the main features of the mitogenomes among subfamilies and the possible evolution processes. The rearrangement of protein-coding genes is reported in the Hydropsychidae for the first time, and it can be explained by the tandem duplication/random loss model. Phylogenetic analyses show that the four monophyletic subfamilies (Arctopscychinae, Diplectroninae, Hydropsychinae, Macronematinae) were strongly supported by mitogenomes.

**Abstract:**

Gene rearrangement of the mitochondrial genome of insects, especially the rearrangement of protein-coding genes, has long been a hot topic for entomologists. Although mitochondrial gene rearrangement is common within Annulipalpia, protein-coding gene rearrangement is relatively rare. As the largest family in Annulipalpia, the available mitogenomes from Hydropsychidae Curtis, 1835 are scarce, and thus restrict our interpretation of the mitogenome characteristic. In this study, we obtained 19 novel mitogenomes of Hydropsychidae, of which the mitogenomes of the genus *Arctopsyche* are published for the first time. Coupled with published hydropsychid mitogenome, we analyzed the nucleotide composition evolutionary rates and gene rearrangements of the mitogenomes among subfamilies. As a result, we found two novel gene rearrangement patterns within Hydropsychidae, including rearrangement of protein-coding genes. Meanwhile, our results consider that the protein-coding gene arrangement of *Potamyia* can be interpreted by the tandem duplication/random loss (TDRL) model. In addition, the phylogenetic relationships within Hydropsychidae constructed by two strategies (Bayesian inference and maximum likelihood) strongly support the monophyly of Arctopscychinae, Diplectroninae, Hydropsychinae, and Macronematinae. Our study provides new insights into the mechanisms and patterns of mitogenome rearrangements in Hydropsychidae.

## 1. Introduction

The mitochondrial genome (mitogenome) of insects is approximately 14,000–20,000 bp in size [1], containing 13 protein-coding genes (PCGs), two ribosomal RNAs (rRNAs), 22 transfer RNAs (tRNAs), and one non-coding control region (CR) [2]. It is characterized by easy availability, maternal inheritance, a low rate of recombination, and a high substitution rate [3,4,5]. Generally, the mitogenome is considered as an effective molecular marker for inferring phylogenetic and evolutionary studies and has been widely used in the studies of speciation [6], phylogeography [7,8,9], and molecular evolution [10]. In addition, the evolution of insect mitogenome rearrangement is also a hot topic [11]. While the typical order of mitochondrial genes is stable in most insects, gene rearrangements of tRNA and PCGs, as well as duplication of tRNA, were also frequently found in Hymenoptera [12,13], Hemiptera [14,15], Thysanoptera [16], Psocodea [17,18], and Coleoptera [19,20], and synapomorphy of the gene rearrangement has been found at different taxonomic levels in multiple insect orders [21,22]. Thus, the gene rearrangement characteristics could also provide effective information for phylogenetic reconstruction [23]. Benefitted by the “next-generation” sequencing technology, the published mitogenomes of Trichoptera, one of the most important aquatic insects and environmental monitoring groups, have rapidly increased [24]. The numerous gene rearrangements in the mitogenome of Annulipalia, including PCGs rearrangement in Polycentropodidae Ulmer, 1903, Ecnomidae Ulmer, 1903, and Pseudoneureclipsidae Ulmer, 1951 were reported. However, the rearrangement patterns of most genera in some families with high species diversity, such as Hydropsychidae, have not been clarified.

**Figure 1:**
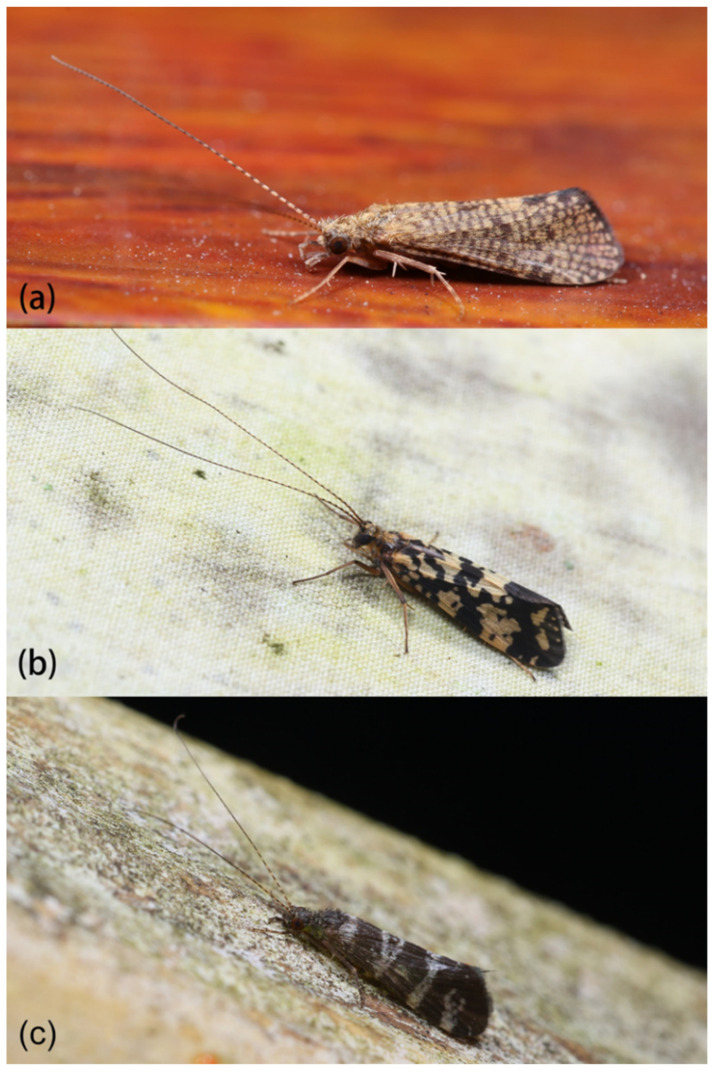
Live adults of hydropsychid species. (**a**) *Hydropsyche* sp., female, from Guangdong, China; (**b**) *Hydromanicus* sp., female, from Guangdong, China; (**c**) *Cheumatopsyche* sp., male, from Guangdong, China ((**a**–**c**) photographed by Qianle Lu).

Containing over 2000 described species over the world, Hydropsychidae Curtis, 1835 (Figure 1) is the largest family in Annulipalpia and is distributed on all continents except Antarctica [25,26]. It consists of five subfamilies: Arctopscychinae Martynov 1924, Diplectroninae Ulmer 1951, Hydropsychinae Curtis 1835, Macronematinae Ulmer 1905, and Smicrideinae Flint 1974 [27,28]. Additionally, its larvae can be found in headwaters, streams, and rivers and they are also bioindicators for monitoring the health of freshwater ecosystems [29]. Compared to its diverse species, the positions of PCGs are rather stable among sequenced mitogenomes of Hydropsychidae; however, rearrangement of tRNA displayed different degrees of variation [24,30]. At the genus level, *Hydromanicus* Brauer, 1865, *Parapsyche* Betten, 1934, *Diplectrona* Westwood, 1840, and *Macrostemum* Kolenati, 1859, translocation of *trnI* was translocated to downstream of *trnQ*. The gene cluster “*trnV-trnQ-trnI-trnM*” was found in *Maesaipsyche* Malicky & Chantaramongkol, 1993. The remote inversion of *trnQ* was considered as a clear molecular synapomorphy for *Cheumatopsyche* Wallengren, 1891 [31]. To date, the mitogenomes of only 16 hydropsychid species have been published, and the comparative analysis of nucleotide composition and evolutionary rates among subfamilies has never been carried out. The situation limits our understanding of the mitogenome characteristics and, furthermore, limits the application of multi-marker DNA metabarcoding technology in water quality monitoring [32].

In addition, the phylogenetic position of five hydropsychid subfamilies has been debated. The hypotheses on the phylogenetic relationship among hydropsychid subfamilies based on morphological characteristics proposed by Ross [33] and Schefter [34] are controversial. Based on the integrated morphology and four molecular markers (mtCOI, 18SrRNA, 28SrRNA, and EF1a), Geraci et al. [26] recovered the monophyly of Arctopsychinae, Macronematinae, and Smicrideinae, but the phylogenetic relationship among the subfamilies remained unclear. More recently, Ge et al. [24] attempted to use the mitogenome to explore the phylogeny of the Trichoptera at the higher categories but were unable to resolve the phylogenetic relationships between the subfamilies because of insufficient sequenced samples of the family. It is unknown whether mitogenomes can be applied to reveal phylogenetic relationships among subfamilies within Hydropsychidae.

In order to understand the mitogenome characteristics and phylogeny of Hydropsychidae, we generated 19 novel mitogenomes of Hydropsychidae species (Table S1), which belong to four subfamilies. Coupled with published partial mitogenomes of Hydropsychidae, we analyzed the main features of the mitogenomes among subfamilies as well as the new rearrangement patterns of genes and possible evolution processes. Finally, we reconstructed the phylogenetic relationships of Hydropsychidae.

## 2. Materials and Methods

### 2.1. Taxon Sampling and DNA Extraction

In total, 18 species were collected using pan traps with 15 w ultraviolet light bulbs in China during 2019–2021. All specimens were preserved in 100% ethanol and stored at −20 °C before morphological examination and DNA extraction. Specimen identifications were made by X-y. Ge, L Peng, and C-h. Sun. The genomic DNA was extracted from the legs using the animal tissue protocol of the Ezup Column Animal Genomic DNA Purification kit (Sangon Biotech, Shanghai, China) according to the manufacturer’s protocol. The vouchers and DNA of the specimen are deposited at the College of Plant Protection, Nanjing Agricultural University, Nanjing, Jiangsu Province, China. The raw data of *Parapsyche elsis* Milne, 1936 were downloaded from Sequence Read Archive (SRA). Detailed taxon sampling information is shown in Table S2. The structural characteristics of mitogenomes were analyzed in combination with 13 published hydropsychid mitogenomes downloaded from GenBank. Based on the phylogenies of Annulipalpia, we selected nine previously reported trichopteran species (one Psychomyiidae species, four Philopotamidae species, three Stenopsychidae species, and one Xiphocentronidae species) as outgroups (Table S1) to reconstruct phylogenetic trees [35].

### 2.2. Amplification and Sequencing 

The mtCOI PCR amplification, fragment sequencing, and analysis followed the procedures of Xu et al. [36]. The primers (LCO1490/HCO2198) are listed in Table S3 [37]. Genomic DNA was sent to Berry Genomics (Beijing, China). The Illumina sequencing libraries with an insert size of 350 bp were constructed for single samples. The libraries conducted paired-end 150 bp sequencing using the Illumina NovaSeq 6000 platform. Each sequencing library produced approximately 4–6 Gb raw data. Trimmomatic v0.32 (Jülich, Germany) [38] was used to remove the adapters and the short and low-quality reads from the raw data. Raw data of *Parapsyche elsis* Milne, 1936 were retained (6 Gb) using BBMap v35.85 [39] for assembling the mitogenome.

### 2.3. Assembly, Annotation and Composition Analyses

To ensure the accuracy of assembly, we used two de novo assembly methods. NOVOPlasty v3.8.3 [40] (Brussel, Belgium) was used to assemble mitogenome with mtCOI sequences as seeds and k-mer sizes of 23–39 bp. IDBA-UD v1.1.3 [41] (Boston, MA, USA) was used for de novo assembly with parameter “--mink 40 --maxk 120”. Geneious 2020.2.1. [42] was used to compare mitogenome sequences obtained by the two methods and merge them into a single sequence. The MITOS2 webserver [43] was used to predict tRNAs and their secondary structure with the invertebrate mitochondrial genetic code. MitoZ v2.4 pipeline (Shenzhen, China) [44] was used to annotate PCGs. The boundaries of rRNAs and PCGs were further proofread using the ClustalW in MEGA X [45]. Nucleotide composition and bias of the nucleotide composition of each gene were calculated using SeqKit v0.16.0 (Chongqing, China) [46]. DnaSP 6.0 (Barcelona, Spain) [47] was used to calculate the rates of non-synonymous substitution rate (Ka)/synonymous substitution rate (Ks) for each PCG. The evolutionary pathways of mitogenome arrangement were predicted using CREx [48] on the web page.

### 2.4. Phylogenetic Analyses

Phylogenetic analyses were conducted based on 13 PCGs and two rRNAs genes of 41 mitogenomes. The nucleotide and protein sequences for each were aligned using L-INS-I algorithm in MAFFT version 7.470 (Osaka, Japan) [49] and trimmed using trimal v1.4.1 (Barcelona, Spain) [50] with “-automated1” strategy. The trimming alignments were then concatenated as five matrixes using FASconCAT-G v1.04 (Santa Cruz, CA, USA) [51]: (1) the PCG_faa matrix containing all PCGs amino acid sequences (3535 sites); (2) the PCG_fna matrix containing all PCGs nucleotide sequences (10,605 sites). (3) the PCG12_fna matrix containing all PCGs nucleotide sequences (with third codon positions removed, 7070 sites); (4) the PCG_rrna matrix containing all PCGs and two rRNA nucleotide sequences (12,564 sites); and (5) the PCG12_rrna matrix containing all PCGs nucleotide sequences (with third codon positions removed) and two rRNA genes (9029 sites). ALIGROOVE (Bonn, Germany) [52] was used to preliminarily analyze the heterogeneity of sequence divergence within five supermatrixs with the default sliding window size. DNA Indels were treated as ambiguity in the nucleotide supermatrixes, and the amino acid substitution matrix with BLOSUM62 matrix.

Each matrix was used to infer the phylogenetic relationships using two different methods, Bayesian inference (BI) and maximum likelihood (ML). For ML analysis, we selected the best-fitting substitution models for each gene partition using MODELFINDER [53] within IQ-TREE v2.0.7 (Canberra, ACT, Australia) [54]. Trees were constructed using IQ-TREE, and 1000 SH-aLRT [55] and UFBoot2 [56] replicates were run for all ML analyses. To reduce the heterogeneous effect, we used the posterior mean site frequency (PMSF) model [57] and general heterogeneous evolution on a single topology (Ghost) model [58] for amino acid and nucleotide, respectively. BI tree was conducted using Phylobayes-MPI v1.8 (Montréal, Canada.) [59], with the site-heterogeneous mixture model CAT + GTR. Two independent Markov chain Monte Carlo chains (MCMC) were carried out and stopped after the two runs had satisfactorily converged (maxdiff < 0.3). The initial 25% trees of each MCMC run were discarded as burn-in, and a consensus tree was calculated from the remaining trees combined. All phylogenetic results were displayed in iTOL version 4 [60] (available at https://itol.embl.de/upload.cgi (accessed on 15 July 2022)).

## 3. Results

### 3.1. Mitogenome General Features of Hydropsychidae

We obtained the novel 19 mitogenomes of hydropsychid species, belonging to four subfamilies and eight genera, from which the mitogenomes of the genus *Arctopsyche* McLachlan, 1868 were reported for the first time. There are 13 complete mitogenomes, and 6 linear mitogenomes (*Arctopsyche spinescens* Gui & Yang, 2001, *Cheumatopsyche* sp., *Hydropsyche columnata* Martynov, 1931, *Macrostemum radiatum* (McLachlan, 1872), *Potamyia chinensis* (Schmid, 1965), and *Potamyia horvati* Malicky & Chantaramongkol, 1997), ranging in length from 14,974 to 27,450 bp. All mitogenomic sequences included 37 canonical mitochondrial genes. Most of the newly obtained mitogenomes were similar to previously reported mitogenomes for Hydropsychidae in length.

**Figure 2:**
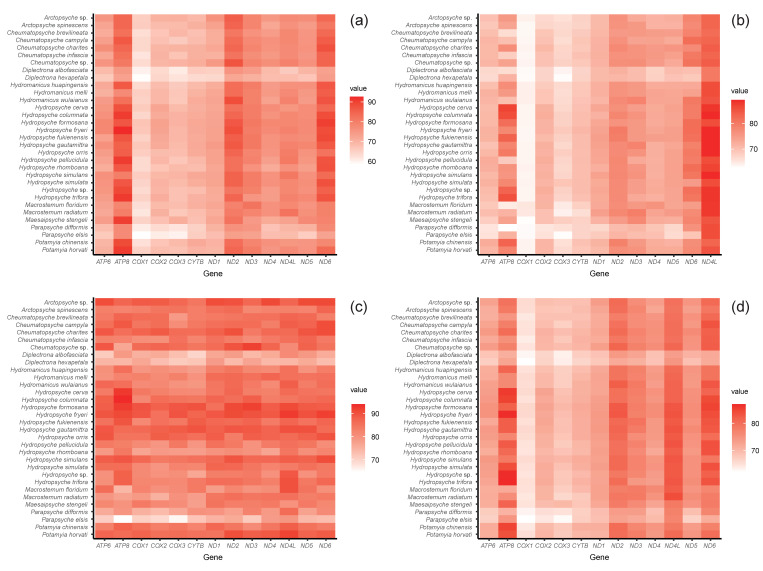
A+T content of protein-coding gene of hydropsychid mitogenomes for different codon positions. The value increases from white to red. (**a**) First codon positions; (**b**) second codon positions; (**c**) third codon positions; (**d**) first/second codon positions.

The mitogenomes exhibited the typical A+T biased composition of insects. The A+T content of the mitochondrial genome ranges from 72.92% to 85.40% (Table S1). The newly obtained mitogenomes had a negative GC-skew. The mitogenome of *Cheumatopsyche* sp. and *Arctopsyche* sp. showed negative AT-skew; this is in contrast with positive AT-skew in other species of the family (Table S4). The 22 typical tRNAs were identified, ranging in length from 56 to 76 bp. The secondary structure of *trnS1* lacks the dihydrouridine (DHU) arm, which is a common characteristic in trichopteran mitogenomes (Figure S1). Combined with previously published hydropsychid mitogenomes, our results show that the A+T content of first and second codon positions was significantly lower than that of third codon positions in the PCGs (Figure 2).

**Figure 3:**
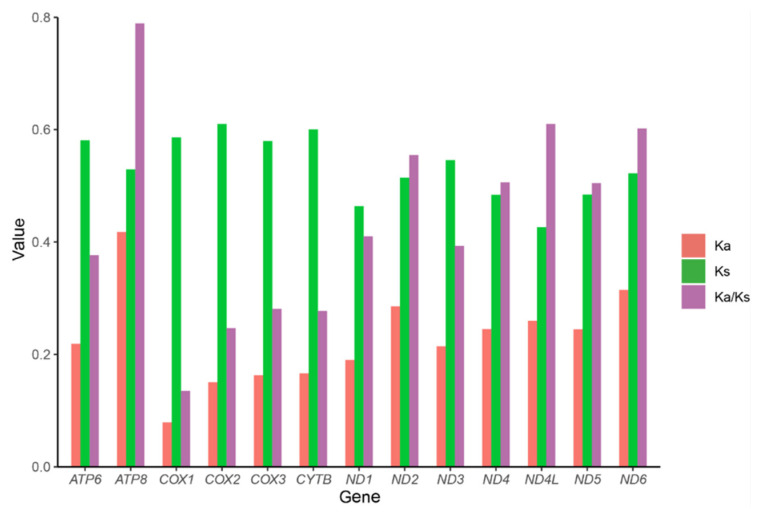
Evolution rate of each protein-coding gene of the hydropsychid mitogenomes. Ka refers to non-synonymous substitution rate, Ks refers to synonymous substitution rate, and Ka/Ks ratio to evolution rate of each protein-coding gene.

The PCG transcribed from the minus strand showed positive GC-skew and negative AT-skew (Figure S2). There was no significant difference in the length of PCG, tRNA and rRNA between different species (Figure S3). The size discrepancy of hydropsychid mitogenome was mainly due to the difference in the size of the control regions and the intergenic spacer (IGS). At the subfamily level, the A+T content of the Hydropsychinae was significantly higher than that of the Diplectroninae (Figure S4; Wilcoxon rank sum test; *p* values ≤ 0.05), while AT-skew and GC-skew values showed no significant difference among subfamilies. Among Hydropsychidae, most PCGs had the typical start codon ATN, and most PCGs had more than three start codons, except for *ATP6* and *COX3*, which started only with ATG (Figure S5). The start codon of *ND1* and *ND5* was in some species TTG, and *COX2* in *Cheumatopsyche brevilineata* and *Cheumatopsyche* sp. started with GTG. *COX2*, *ND5*, and *ND1* in most Hydropsychid species have an incomplete termination codon TA or T. The average ratio of Ka/Ks (ω) was used to investigate for the signatures of natural selection. The Ka/Ks values of 13 PCGs were less than 1.0, ranging from “0.7893 (ATP8) to 0.1351 (COX1)” Figure 3). Different genes were under different states of purifying pressures, of which *ATP8*, *ND4L*, and *ND6* exhibited relatively relaxed purifying selection. DNA barcoding gene *COX1* was under the strongest purifying selection, which is consistent with previous studies of Trichoptera. At the subfamily level, the (ω) value of Diplectroninae is significantly lower than those of the other three subfamilies, indicating each gene of Diplectroninae undergoes the most severe purifying selection (Figure S6).

### 3.2. Gene Rearrangement of Hydropsychidae

**Figure 4:**
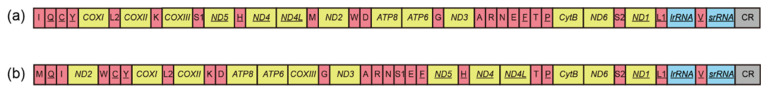
The mitochondrial gene order of the genera *Potamyia* and *Arctopsyche*. Genes are transcribed from left to right except those underlined, which have the opposite transcriptional orientation. Protein-coding genes are indicated in yellow, tRNA in red, rRNA genes in blue, and control regions in gray: (**a**) *Potamyia chinensis* and *Potamyia horvati*; (**b**) *Arctopsyche spinescens* and *Arctopsyche* sp.

In our results, we revealed novel PCGs rearrangements in two species of the genus *Potamyia* Banks, 1900 by two de novo assembly methods; seven of the 13 PCGs and eight tRNAs had changed positions (Figure 4a). The gene rearrangement occurred in the gene cluster “*trnM* to *ND4L*”, and the genes of the same polarity were not rearranged to a cluster, which differs from those previously reported rearrangements of PCGs. In the absence of sequence information on more *Potamyia* species, it is unclear whether this rearrangement event is common in the genus *Potamyia*. The tRNA rearrangement is common in Annulipalpia. Accordingly, the gene cluster “*trnI-trnQ-trnM*” was also considered as the frequent rearrangement region in the Hydropsychidae. Previously, the rearrangement pattern of the “*trnQ-trnI-trnM*” universally occurred in five sequenced genera (*Cheumatopsyche*, *Diplectrona*, *Parapsyche*, *Hydromanicus*, and *Macrostemum*). Our results showed that the tRNA rearrangement patterns of the newly sequenced species of the above five genera were consistent with those of published species of these genera. The novel gene rearrangement “*trnM-trnQ-trnI*” was found in the genus *Arctopsyche* (Figure 4b).

### 3.3. Phylogenetic Relationships

The heterogeneous sequence divergence analysis of each supermatrix for taxa indicated that Macronematinae and Diplectroninae exhibited higher heterogeneity than other subfamilies, and the lowest heterogeneity was found in Hydropsychinae (Figure S7). The heterogeneity of PCG_faa, PCG12_fna, and PCG12_rrna datasets was lower than that of PCG_fna and PCG_rrna datasets. Due to the high heterogeneity of the third codon positions, we excluded the third codon during phylogenetic reconstruction.

**Figure 5:**
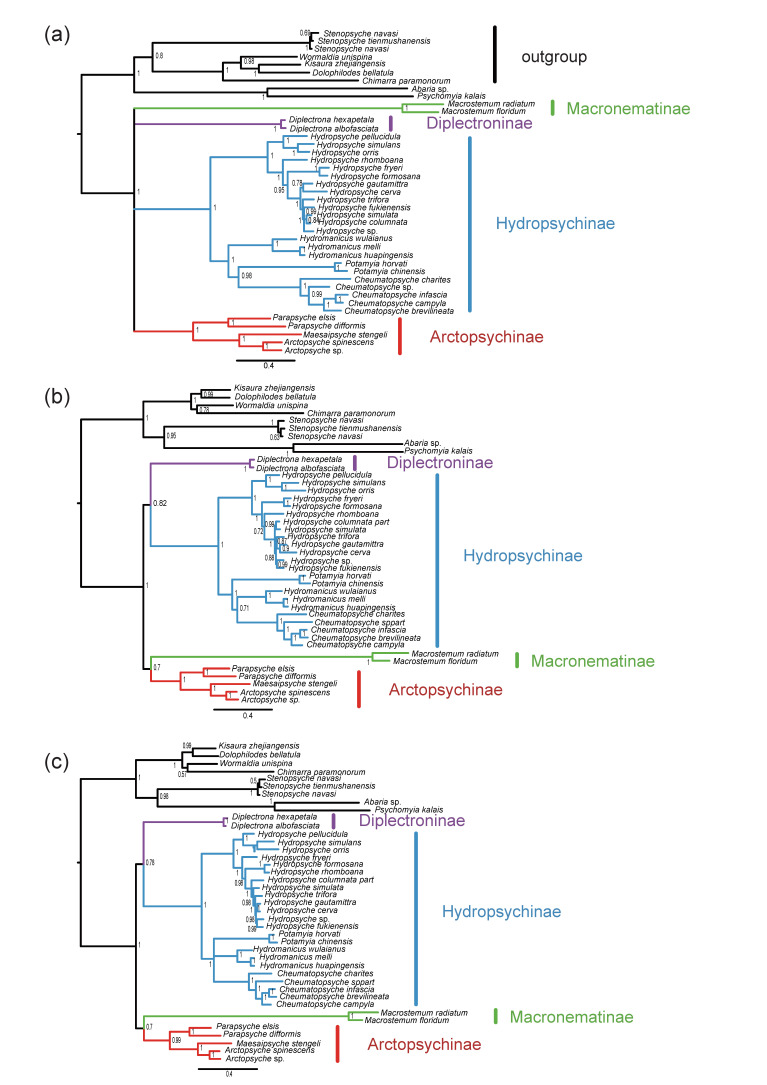
Phylogeny of Hydropsychidae based on three datasets using GTR+CAT mode in phylobayes. Node supports are Bayesian posterior probabilities. (**a**) PCGs_faa dataset; (**b**) PCG12_fna dataset; (**c**) PCG12_rrna dataset.

In this study, three datasets (PCG12_fna, PCG12_rrna, and PCG_faa) were used to explore the phylogenetic relationships of Hydropsychidae by two strategies, which produced two different topologies. The monophyly of Hydropsychidae and four subfamilies (Arctopsychinae, Hydropsychinae, Diplectroninae, and Macronematinae) were recovered, but phylogenetic relationships were not well supported between each subfamily. In the BI, four subfamilies formed the topology of (Hydropsychinae + Diplectroninae) + (Arctopsychinae + Macronematinae) (Figure 5), which were also found that the ML tree of PCG_faa datasets (Figure S8), while ML analysis of PCG12_fna and PCG12_rrna showed that (Macronematinae + (Hydropsychinae + (Diplectroninae + Arctopsychinae))) (Figures S9 and S10).

## 4. Discussion

### 4.1. Mitogenome Features of Hydropsychiae

A total of 19 mitogenomes of Hydropsychidae are included in our study, of which 13 are complete mitogenomes and 6 are linear mitogenomes. The nucleotide composition of the mitogenomes of Hydropsychid species is biased toward A+T, which is consistent with other published trichorpteran species. Their composition skew values are significantly different from PCGs transcribed from the plus strand, which is consistent with the previously reported mitochondrial characteristics of Trichoptera [24]. Most PCGs of hydropsychid species terminated with complete termination codons, while some PCGs had an incomplete termination codon TA or T, which is associated with post-transcriptional modification during mRNA maturation [61].

### 4.2. Gene Rearrangement

**Figure 6:**
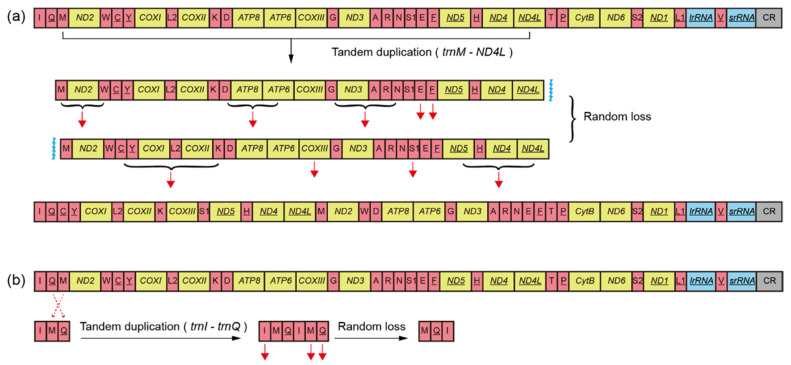
The mechanism proposed for gene rearrangement in *Potamyia* and *Arctopsyche* by tandem duplication and random loss model. The colors of the gene and control region are the same as in Figure 5. The black arrow indicates gene tandem-duplication. The red dotted arrow indicates the absence of a gene. (**a**) the genus *Potamyia*; (**b**) the genus *Arctopsyche*.

As indicated by a previous study, gene rearrangement is a common phenomenon found in the mitogenomes of Trichoptera, especially in Annulipalpia [24]; moreover, tRNA rearrangements were found in almost all the sequenced hydropsychid species. However, PCGs rearrangement events were rare in insects. They have been reported in Thysanoptera [62], Psocodea [17], Hemiptera [63], and Hymenoptera [12]. In Trichoptera, PCGs rearrangement, as synapomorphy within the family, was found only in Ecnomidae, Polycentropodiae, and Pseudoneureclipsidae but has never been reported in the Hydropsychidae. Ge et al. [24] summarized five rearrangement patterns of genes from 13 species in the Hydropsychidae, and they found that the gene clusters “*trnI* to *trnM*”, “*trnT*-*trnP*” and “*ND1* to *srRNA*” were the “hot spot” regions of gene rearrangement in Hydropsychidae. Tandem duplication/random loss (TDRL) [64] and tandem duplication/nonrandom loss (TDNL) [65] were often used to explain the mechanism of PCGs rearrangement. The PCGs rearrangement of Ecnomidae, Polycentropodiae, and Pseudoneureclipsidae was thought to be caused by a tandem duplication/nonrandom loss event. Based on the CREx of analysis and position of IGS, we hypothesize that it could probably be explained as follows. Firstly, the gene tandem duplication occurs in the gene cluster “*trnM* to *ND4L*” and generates two sets of the same gene region. Secondly, the supernumerary gene is then eliminated, resulting in the present pattern (Figure 6a). In the process of gene loss, half of the genes are lost in each of the two copies of the gene cluster. Therefore, we consider that the loss of genes in the second stage is random. PCGs rearrangement of the genus *Potamyia* could be the result of TDNL events. In the future, mitochondrial sequencing of more *Potamyia* species could allow us to clarify gene rearrangement rules in the genus. The novel gene rearrangement “*trnM-trnQ-trnI*” has previously been observed also in Hymenoptera (Cephidae) [6,66]. Based on the *trnI* downstream intergenic spaces (IGSs), we consider that the gene cluster probably underwent two round TDRL leading to the present pattern (Figure 6b). In addition, we also find the gene rearrangement pattern “*trnT-ND2-trnS2-trnP*” in the novel sequenced mitogenome of *Hydropsyche* Pictet, 1834.

The numerous gene rearrangements are found in the hydropsychid mitogenome by de novo assembly. Nevertheless, we did not find gene rearrangement in the six mitogenomes of the Hydropsychidae published by Marcus [67], which hindered us from using rearrangement as an effective marker for the phylogeny of Hydropsychidae. Comparing with the methods of Marcus, we found that six hydropsychid mitogenomes were assembled using reference mitogenomes in their result, but the reference mitogenomes they used did not have gene rearrangement belonging to Integripalpia [68,69]. We speculated that the structure of the reference genome and the method of assembly influence the assembly result of the mitogenome. To test our hypothesis, we used the reference assembly method to assemble mitogenome using the raw data of the genera *Hydropsyche* and *Potamyia* in our study. *Anabolia bimaculata* (Walker, 1852) and *Triaenodes tardus* Milne, 1934 were selected as reference mitogenomes. The results display that the gene order mitogenome using the reference assembly method consists of that of the reference sequence, which confirms our speculation. Since we are unable to obtain the raw data from previous studies, we need to collect samples of these species for future studies to further confirm our viewpoint.

There are plentiful gene rearrangements and relatively high A + T content in the Hydropsychidae; thus, we suggest that the hydropsychid mitogenome can be accurately obtained by the strategy of the de novo assembly and the construction of the single-sample library. With the increasing number of mitogenomes in Hydropsychidae, abundant gene rearrangements will be found in the mitogenome. The gene order rules are becoming more explicit at the genus level. In general, the gene rearrangement may be the result of the rapid evolution of this group. Species within the same genus tend to exhibit identical gene orders, which indicates that gene rearrangements may be useful for phylogenetic analysis between genera.

### 4.3. Phylogenetic Analyses

In our study, we obtained two topologies, which are phylogenetic relationships among subfamilies that are contradictory with systematics based on traditional morphology and a few DNA markers [26,35,70]; meanwhile, the phylogenetic relationship using different strategies among subfamilies is also erratic. Even though the heterogeneity model (CAT + GTR) is used to reconstruct phylogenetic tree, the phylogenetic relationships among subfamilies are not well supported (posterior probabilities (PPs) < 95). We believe that this result is due to the high nucleotide substitution rate of Hydropsychidae and the absence of sequenced data of the Smicrideinae. Previous studies indicated that missing taxons, lack of informative genetic characters, and higher nucleotide substitution rates lead to errors in phylogenetic estimates [71]. Therefore, we suggest that the mitogenome cannot be used to resolve the phylogenetic relationship at the subfamily level in Hydropsychidae. To comprehend the evolutionary history of the Hydropsychidae, we still need a more comprehensive sampling and more molecular markers, i.e., single-copy orthologous genes or ultra-conserved elements [72].

## 5. Conclusions

The present study obtained 19 mitogenomes of hydropsychid species. Coupled with published hydropsychid mitogenomes, we performed analyses of base composition, gene rearrangement, and phylogenetic relationships within Hydropsychidae. The gene rearrangements were found in all the newly obtained mitogenomes. Simultaneously, two new rearrangement patterns were found in the genera *Potamyia* and *Arctopsyche*, and the novel two gene rearrangement patterns perhaps were due to one or more TDRL events at different scales. The phylogenetic analysis strongly confirmed the monophyly of Hydropsychidae. Our results provide new insight into the exploration of mitogenomic evolution in Hydropsychidae.

## Data Availability

The voucher specimens from this research were deposited in the Insect Classification and Aquatic Insect Laboratory, College of Plant Protection, Nanjing Agricultural University, Nanjing, China. The newly assembled sequences accession number are available in Table S1. The newly sequenced mitogenomes are available at NCBI (BioProject ID: PRJNA869364).

## Supplementary Materials

The following supporting information can be downloaded at: https://www.mdpi.com/article/10.3390/insects13090759/s1. Table S1: Detailed taxonomic resources are used in the present study. Table S2: Collection of information of the newly sequenced samples. Table S3: PCR primers were used to sequence mtCOI genes of Hydropsychidae in this study. Table S4: The length, nucleotide composition, and skewness of novel obtained hydropsychid mitogenomes. Figure S1: Putative secondary structures of the 22 tRNA genes identified in the mitogenome of Hydropsyche cerva Li & Tian, 1990. Figure S2: Box-and-whisker plots for nucleotide composition of each gene. (a) AT-skew; (b) GC-skew. Figure S3. The length of protein-coding genes, transfer RNAs, ribosomal RNAs, and control regions among 32 hydropsychid mitogenomes. Figure S4: A+T content of each subfamily. Asterisks indicate *p* values ≤ 0.05 (Wilcoxon rank sum test *). Figure S5: Start codons of protein-coding genes among hydropsychid mitogenomes Figure S6. Evolution rate of each PCG of the mitogenomes of four subfamilies. Figure S7: Heterogeneity of sequence composition of mitochondrial genomes for different datasets. The pairwise Aliscore values are represented by colored squares. The scores range from −1, indicating full random similarity (dark blue), to +1, indicating non-random similarity (bright orange). Figure S8: ML phylogenomic tree of Hydropsychidae based on the analysis of PCG_faa dataset with PMSF model in IQ-TREE. Node values represent SH-aLRT and UFBoot2, respectively. Figure S9: ML phylogenomic tree of Hydropsychidae based on the analysis of PCG12_fna dataset with partitioning model in IQ-TREE. Node values represent SH-aLRT and UFBoot2, respectively. Figure S10: ML phylogenomic tree of Hydropsychidae based on the analysis of PCG12_rrna dataset with partitioning model in IQ-TREE. Node values represent SH-aLRT and UFBoot2, respectively.
