# Supplementary material for: Comparative Mitogenomic Analyses of Hydropsychidae Revealing the Novel Rearrangement of Protein-Coding Gene and tRNA (Trichoptera: Annulipalpia)
Source: Insects. 2022 Aug 23;13(9):759. doi: 10.3390/insects13090759 (PMC9501032; doi:10.3390/insects13090759)
Supplement: Supplementary file 1 [file insects-13-00759-s001.zip › insects-1873178-supplementary.pdf]

Table S1. Detailed taxonomic resources are used in the present study.

Subfamily	Subfamily	Genus	Species	GenBank accession number
Ingroup				
Hydropsychidae	Arctopsychinae	*Arctopsyche*	*Arctopsyche spinescens* Gui & Yang, 2001	ON859124
			Arctopsyche sp.	ON859111
		Maesaipsyche	*Maesaipsyche stengeli* Malicky, 1997*	OL677993
		Parapsyche	*Parapsyche difformis* (Banks, 1947)*	OL677995
			Parapsyche elsis Milne, 1936	ON859123
	Diplectroninae	*Diplectrona*	*Diplectrona albofasciata* (Ulmer, 1913)	ON859114
			Diplectrona hexapetala Sun, 2012*	OL678009
	Hydropsychinae	*Cheumatopsyche*	*Cheumatopsyche brevilineata* (Iwata, 1927)	ON859112
			Cheumatopsyche campyla Ross, 1938*	NC_036954
			Cheumatopsyche charites Malicky & Chantaramongkol, 1997*	OL678007
			Cheumatopsyche infascia Martynov, 1934	ON859113
			Cheumatopsyche sp.	ON859125
		Hydromanicus	*Hydromanicus huapingensis* (Li & Tian, 1990)	ON859115
			Hydromanicus melli (Ulmer, 1926)*	OL678021
			Hydromanicus wulaianus (Kobayashi, 1987)*	NC_036156
		Hydropsyche	*Hydropsyche cerva* (Li & Tian, 1990)	ON859117
			Hydropsyche columnata Martynov, 1931	ON859126
			Hydropsyche formosana Ulmer, 1911	ON859118
			Hydropsyche fryeri Ulmer, 1915*	NC_060325
			Hydropsyche fukienensis Schmid, 1965*	OL678022
			Hydropsyche gautamittra Schmid, 1961	ON859119
			Hydropsyche orris Ross, 1938*	NC_036951
			Hydropsyche rhomboana Martynov, 1909	ON859120

			Hydropsyche simulans Ross, 1938*	NC_036950
			Hydropsyche simulata Mosely, 1942	ON859121
			Hydropsyche trifora (Li & Tian, 1990)	ON859122
			Hydropsyche pellucidula (Curtis, 1834)*	NC_029246
			Hydropsyche sp.	ON859116
		Potamyia	*Potamyia chinensis* (Ulmer, 1915)	ON859128
			Potamyia horvati Malicky & Chantaramongkol, 1997	ON859129
	Macronematinae	*Macrostemum*	*Macrostemum floridum* (Navás, 1929)*	MT677867
			Macrostemum radiatum (McLachlan, 1872)	ON859127
Outgroup				
Philopotamidae	Chimarrinae	*Chimarra*	*Chimarra paramonorum* Hu, Sun &Wang, 2018*	OL678008
	Philopotaminae	*Dolophilodes*	*Dolophilodes bellatula* Sun & Malicky, 2002*	OL678011
		Kisaura	*Kisaura zhejiangensis* Hu, 2019*	OL678025
		Wormaldia	*Wormaldia unispina* Sun, 1998*	OL678056
Stenopsychidae	Stenopsychinae	*Stenopsyche*	*Stenopsyche angustata* Martynov, 1930*	NC_051529
			Stenopsyche navasi Ulmer, 1926*	OL678052
			Stenopsyche tienmushanensis Hwang, 1957*	MW201980
Psychomyiidae	Psychomyiinae	*Psychomyia*	*Psychomyia kalais* Malicky, 2004*	OL678047
Xiphocentronidae	Xiphocentroninae	*Abaria*	*Abaria* sp.*	OL677997

* Published mitogenomes of Trichoptera.

Table S2. Collection information of the newly sequenced samples.

Subfamily	Species	Geographical locality	Longitude	Latitude	Elevation (m)	Collection date
Arctopsychinae	*Arctopsyche* sp.	Yunxi, Yunnan, China	101°59′59″E	23°37′1″N	1,624	13.VII.2021
	Arctopsyche spinescens	Menyuan, Qinghai, China	101°36′55″E	37°21′37″N	2,782	30.VI.2021
Diplectroninae	*Diplectrona albofasciata*	Mangshan, Hunan, China	112°55′39″E	24°58′48″N	731	02.IX.2020
	Cheumatopsyche brevilineata	Yueqing, Zhejiang, China	120°59'24"E	28°17'59"N	64	03-13.IV.2019
	Cheumatopsyche infascia	Mentougou, Beijing, China	115°50′21"E	40°1′57"N	408	01.XI.2020
	Cheumatopsyche sp.	Nanjing, Jiangsu, China	118°50′52"E	32°2′40"N	406	05.VI.2021
Hydropsychinae	*Hydromanicus huapingensis*	Huanping, Guanxi, China	109°54′38"N	25°36′5"N	612	19.V.2021
	Hydropsyche cerva	Zhaotong, Yunnan, China	103°1'28"E	26°47'14"N	738	23.XI.2019
	Hydropsyche columnata	Zhaotong, Yunnan, China	103°1'28"E	26°47'14"N	738	23.XI.2019
	Hydropsyche formosana	Wuyishan, Fujian, China	117°37'17"E	27°38'53"N	550	16.III.2020
	Hydropsyche gautamittra	Pingshan, Sichuan, China	103°45'58″E	28°41'8″N	368	19.XI.2019
	Hydropsyche rhomboana	Qilianshan, Qinghai, China	101°36'29"E	37°21'9"N	2,786	29.VII.2020
	Hydropsyche simulate	Shaoyang, Hunan, China	110°22′15"E	26°14′39"N	559	20.V.2021
	Hydropsyche trifora	Wuyishan, Fujian, China	117°37'17"E	27°38'53"N	550	16.III.2020
	Hydropsyche sp.	Shaoyang, Hunan, China	110°22′15"E	26°14′39"N	559	20.V.2021
	Potamyia chinensis	Yueqing, Zhejiang, China	120°59'24"E	28°17'59"N	64	03-13.IV.2019
	Potamyia horvati	Lincang, Yunnan, China	99°31'45"E	23°37'8"N	1,085	29.III.2020
Macronematinae	*Macrostemum radiatum*	Changde, Hunan, China	113°19'24"E	28°17'59"N	63	03-13.IV.2019

Table S3. PCR primers were used to sequence mtCOI genes of Trichoptera in this study.

Primer	Sequence	Reference
LCO1490	GGTCAACAAATCATAAAGATATTGG	Folmer et al.1994
HCO2198	TAAACTTCAGGGTGACCAAAAAATCA	

Table S4. The length, nucleotide composition, and skewness of novel obtained hydropsychid mitogenomes.

Species	length	A+T (%)	AT-Skew	GC-Skew
Arctopsyche spinescens Gui & Yang, 2001	16,175	79.24	0.0492	-0.3329
Arctopsyche sp.	15,923	81.42	-0.008	-0.2592
Parapsyche elsis Milne, 1936	15,931	73.09	0.0157	-0.3688
Diplectrona albofasciata (Ulmer, 1913)	15,566	72.92	0.0494	-0.3716
Cheumatopsyche brevilineata (Iwata, 1927)	15,330	79.1	0.0111	-0.3315
Cheumatopsyche infascia Martynov, 1934	15,275	78.99	0.0168	-0.3375
Cheumatopsyche sp.	14,974	80.41	-0.0097	-0.3003
Hydromanicus huapingensis (Li & Tian, 1990)	15,378	77.79	0.0318	-0.3511
Hydropsyche cerva (Li & Tian, 1990)	17,038	80.61	0.0195	-0.2988
Hydropsyche columnata Martynov, 1931	15,614	80.44	0.0226	-0.2874
Hydropsyche formosana Ulmer, 1911	16,139	82.35	0.0222	-0.2322
Hydropsyche gautamittra Schmid, 1961	15,545	81.54	0.0086	-0.2318
Hydropsyche rhomboana Martynov, 1909	15,507	77.7	0.0152	-0.3169
Hydropsyche simulata Mosely, 1942	18,380	81.51	0.0168	-0.279
Hydropsyche trifora (Li & Tian, 1990)	15,997	79.91	0.0274	-0.2941
Hydropsyche sp.	15,652	78.72	0.0333	-0.3129
Potamyia chinensis (Ulmer, 1915)	22,920	81.75	0.0368	-0.3808
Potamyia horvati Malicky & Chantaramongkol, 1997	27,450	85.4	0.0012	-0.3207
Macrostemum radiatum (McLachlan, 1872)	15,796	78.18	0.0098	-0.415

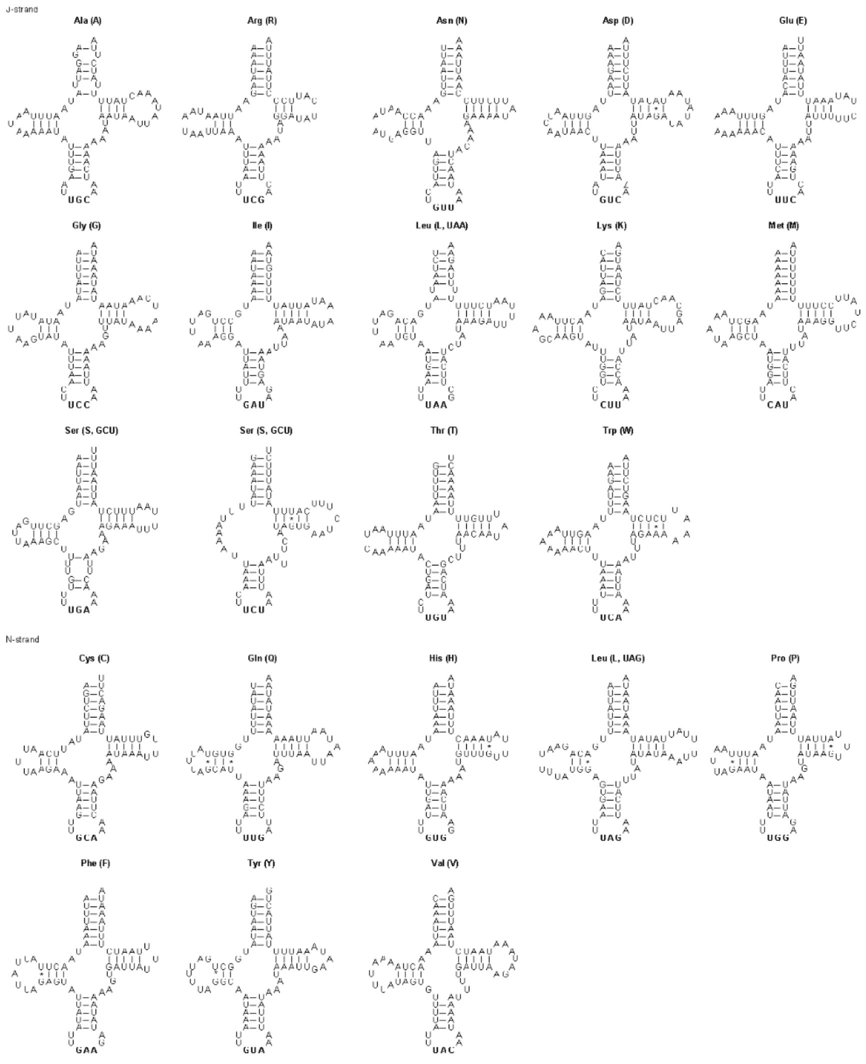

Figure S1. Putative secondary structures of the 22 tRNA genes identified in the mitogenome of *Hydropsyche cerva* Li & Tian, 1990.

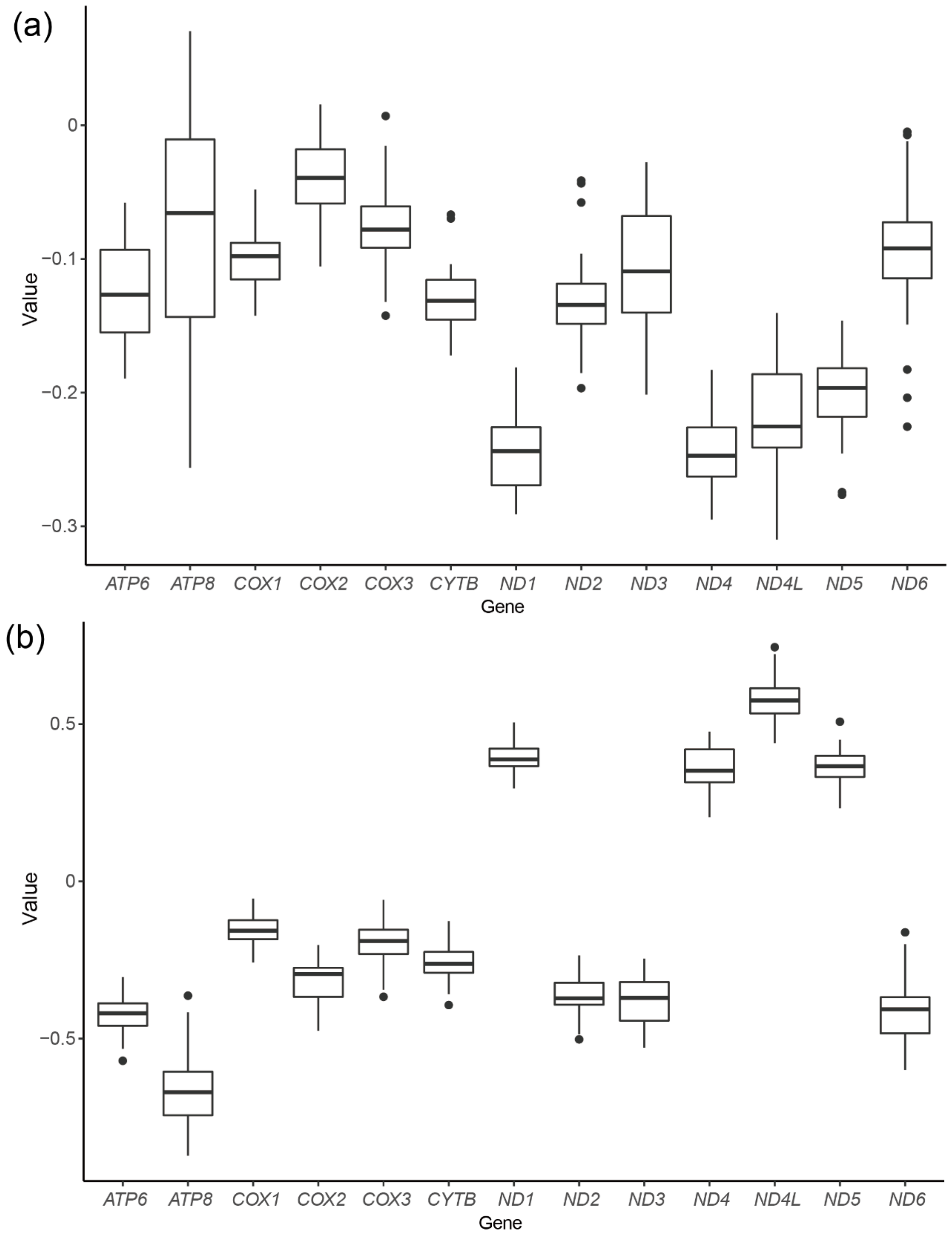

Figure S2. Box-and-whisker plots for nucleotide composition of each gene. (a) AT-skew; (b) GC-skew.

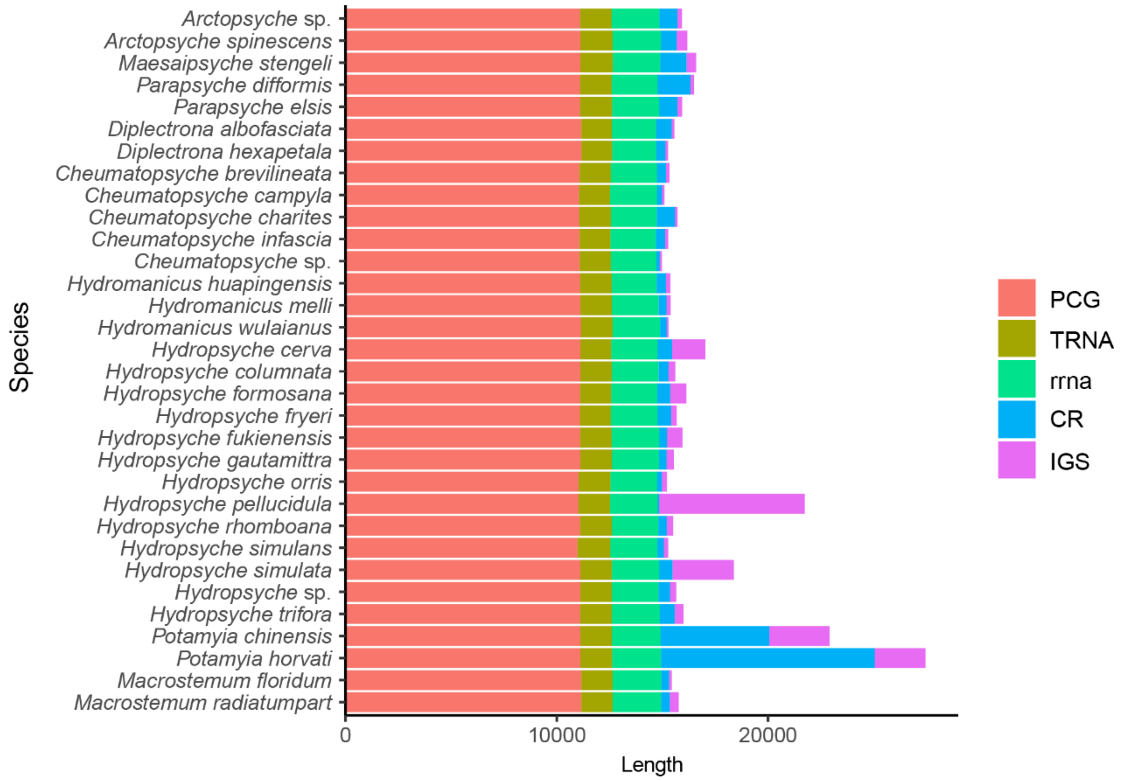

Figure S3. The length of protein-coding genes, transfer RNAs, ribosomal RNAs, and control regions among 32 hydropsychid mitogenomes.

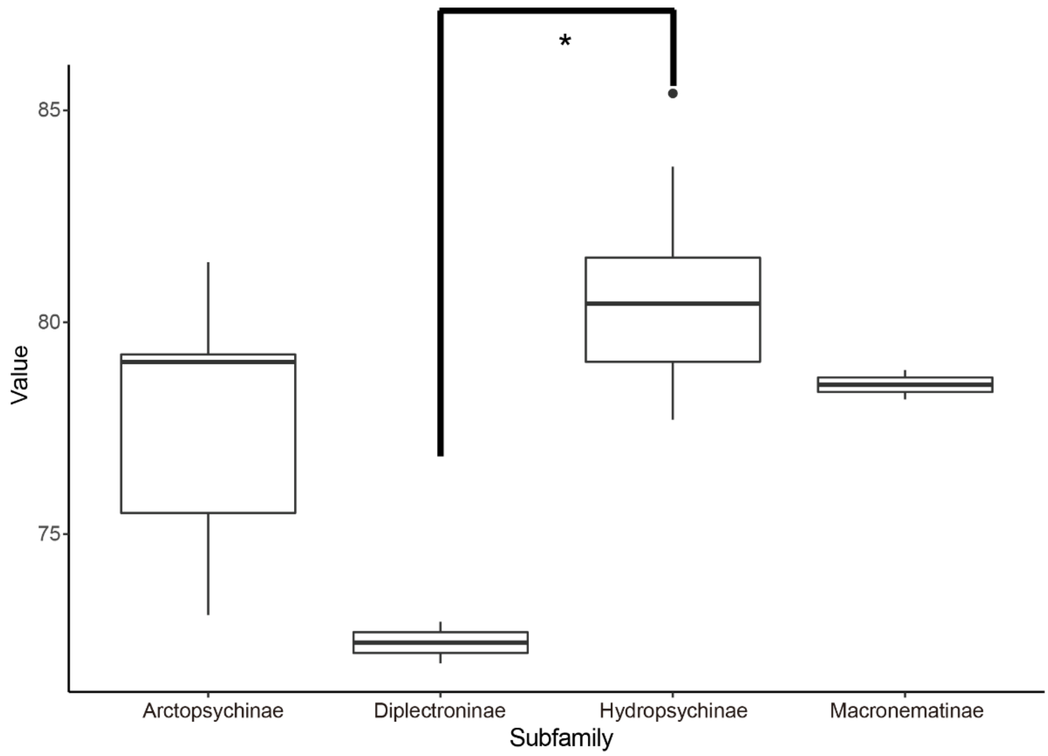

Figure S4. A+T content of each subfamily. Asterisks indicate p values ≤ 0.05 (Wilcoxon rank sum test *).

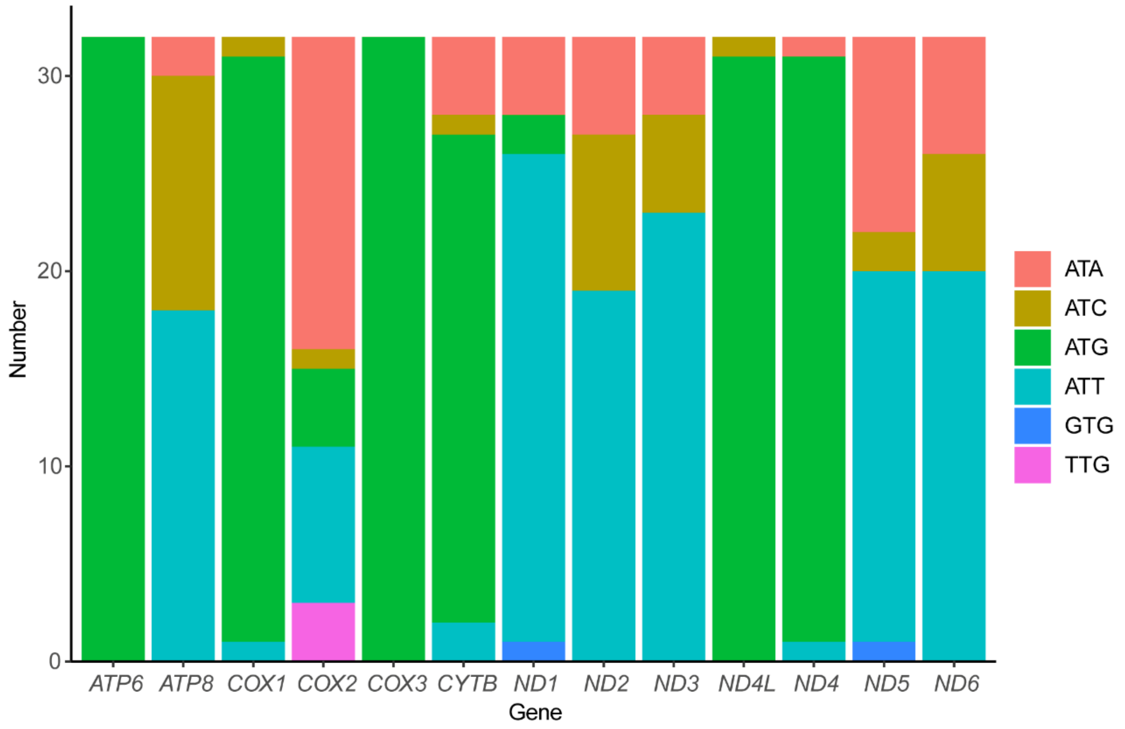

Figure S5. Start codons of protein-coding genes among hydropsychid mitogenomes.

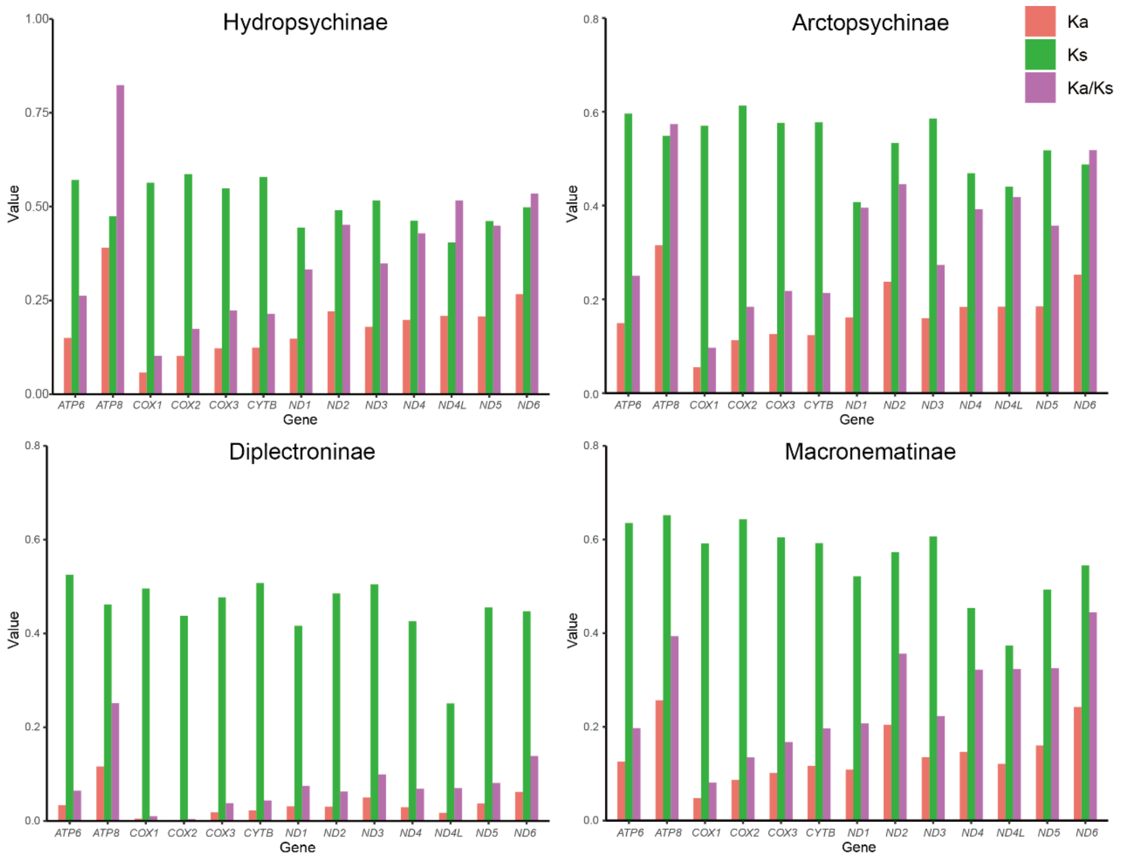

Figure S6. Evolution rate of each PCG of the mitogenomes of four subfamilies.

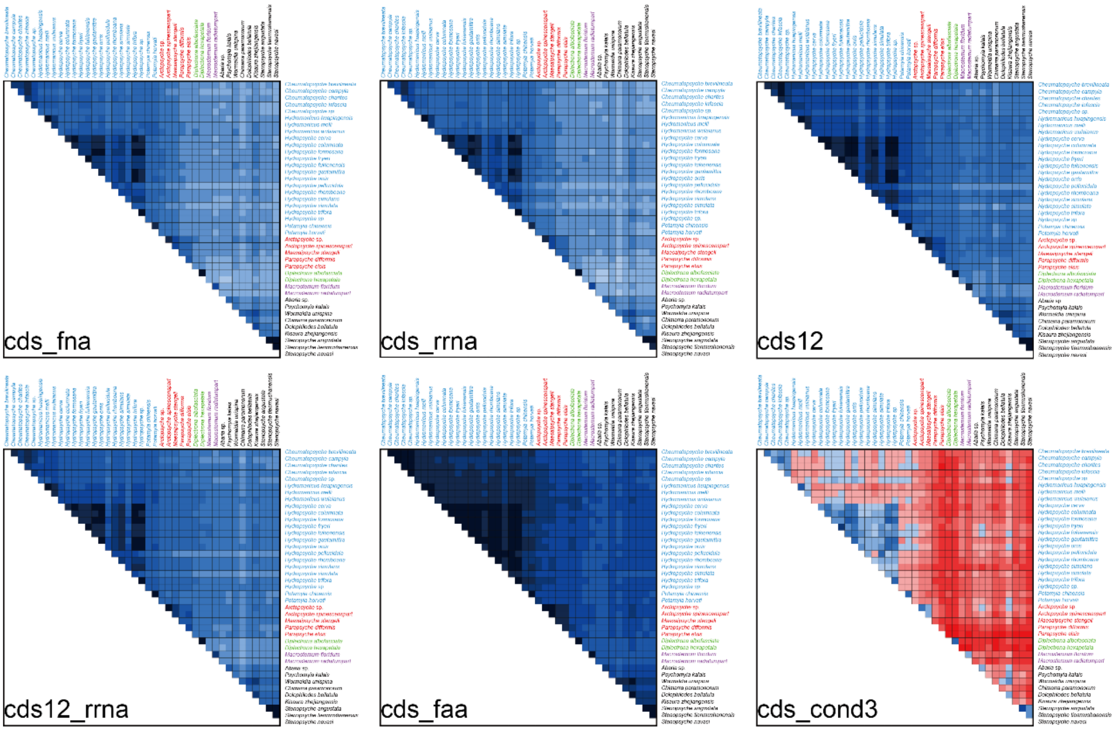

Figure S7. Heterogeneity of sequence composition of mitochondrial genomes for different datasets. The pairwise Aliscore values are represented by colored squares. The scores range from −1, indicating full random similarity (dark blue), to +1, indicating non-random similarity (bright orange).

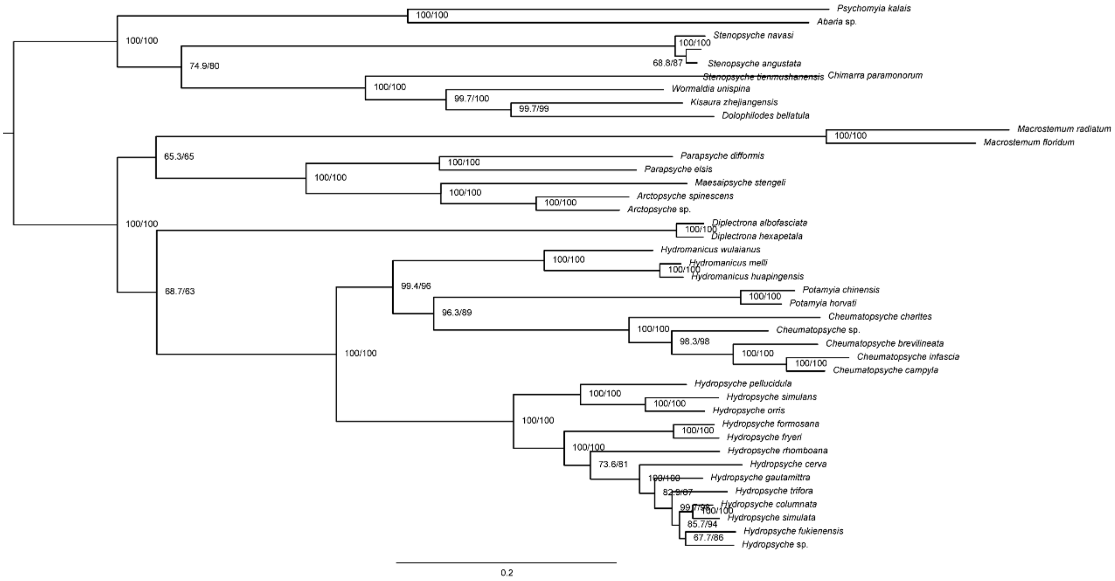

Figure S8. ML phylogenomic tree of Hydropsychidae based on the analysis of PCG_faa dataset with PMSF model in IQ-TREE. Node values represent SH-aLRT and UFBoot2, respectively.

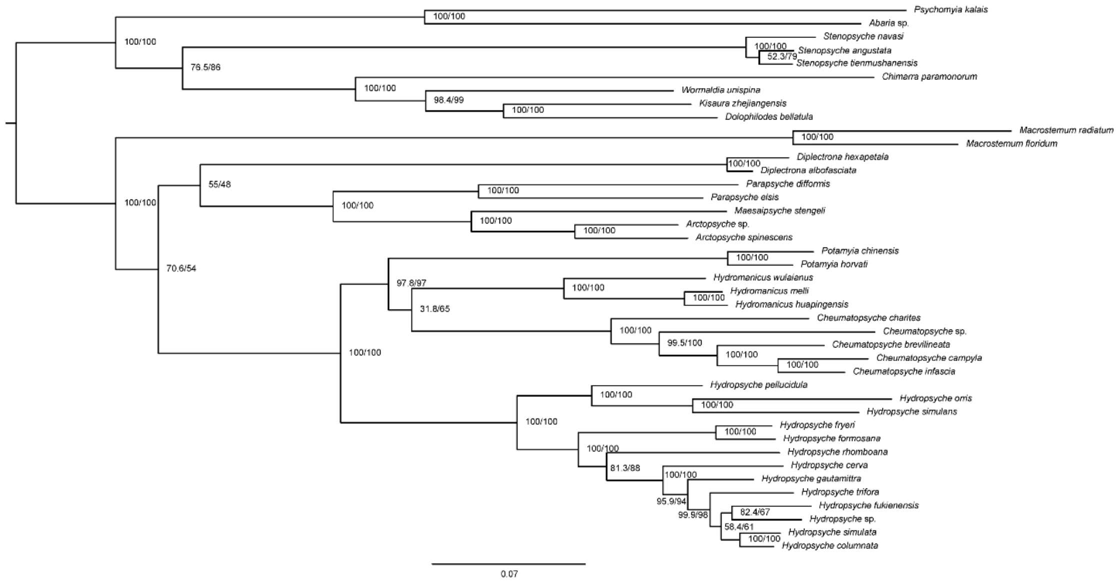

Figure S9. ML phylogenomic tree of Hydropsychidae based on the analysis of PCG12_fna dataset with partitioning model in IQ-TREE. Node values represent SH-aLRT and UFBoot2, respectively.

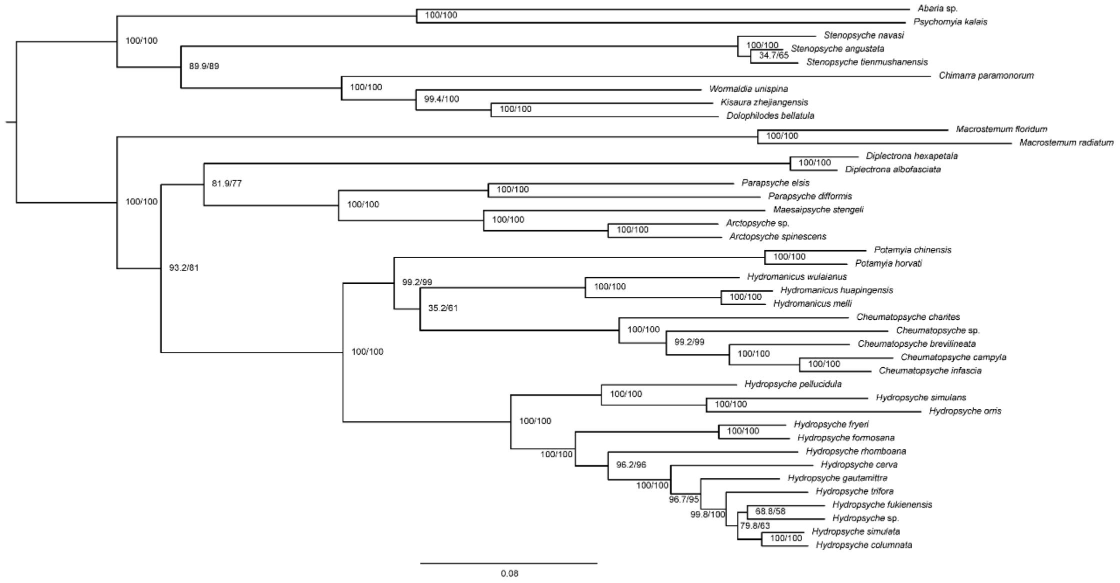

Figure S10. ML phylogenomic tree of Hydropsychidae based on the analysis of PCG12_rrna dataset with partitioning model in IQ-TREE. Node values represent SH-aLRT and UFBoot2, respectively.
